# Preventing Bias in Cluster Randomised Trials

**DOI:** 10.1371/journal.pmed.1000065

**Published:** 2009-05-05

**Authors:** Bruno Giraudeau, Philippe Ravaud

**Affiliations:** 1 INSERM, CIC 202, Tours, France; 2 Université François Rabelais, Tours, France; 3 CHRU de Tours, Tours, France; 4 INSERM, U738, Paris, France; 5 Assistance Publique–Hôpitaux de Paris, Hôpital Bichat, Département d'Epidémiologie, Biostatistique et Recherche Clinique, Paris, France; 6 Université Paris 7–Denis Diderot, Paris, France

## Abstract

Bruno Giraudeau and Philippe Ravaud discuss the difficulties in preventing selection bias and applying intention-to-treat analysis in cluster randomized trials, and propose some solutions.

**Summary Points**

- In CRTs, the comparability of groups is challenged because groups of trial participants rather than the participants themselves are randomised.
- The specific chronology of such trials compromises allocation concealment (i.e., clusters are recruited and randomised and then participants are recruited), which can induce differential recruitment and thus quantitative and qualitative imbalance between groups.
- The principle of intention to treat is challenged in CRTs because of the lack of any statistical method to handle non-recruited participants.
- Empty clusters (i.e., clusters with no data for participants), which are randomised units, are discarded from the analysis—a violation of the very principle of intention to treat.
- Some CRTs may be better analysed as observational studies, with some form of adjustment used such as propensity-score methods.

In randomised trials, internal validity is defined as “the extent to which the design and conduct of a study are likely to have prevented bias” [1],[2]. In conducting such trials, trialists try to prevent selection bias through randomisation and allocation concealment (defined as “the process used to ensure that the person deciding to enter a participant into a randomised controlled trial does not know the comparison group into which that individual will be allocated” [2]) and attrition bias through an intention-to-treat (ITT) analysis. ITT analysis has indeed been defined as one of the cornerstones of the analysis strategy of randomised trials because it allows for preserving the benefits of randomisation [3]. With an ITT analysis, data for all randomised participants are analysed in the groups to which they were originally randomly allocated “regardless of their adherence with the entry criteria, regardless of the treatment they actually received, and regardless of subsequent withdrawal from treatment or deviation from the protocol” [4]. ITT analysis entails the use of ad hoc statistical methods to handle missing outcome data when participants withdraw from the trial or are lost to follow-up [4],[5]. The ITT principle is widely used in analysing data from individually randomised trials but is much more difficult in cluster randomised trials (CRTs), and this issue is not clearly covered in the main methodological textbooks on the topic [6],[7].

Here, we describe the difficulties in preventing selection bias and applying ITT analysis in CRTs (other biases are discussed in Puffer et al. [8]) and propose some solutions to deal with these issues in this trial design.

## CRTs: Randomising Clusters Rather Than Individuals

In CRTs, “intact social units, or clusters of individuals, rather than individuals themselves, are randomised” [6]. For example, hospitals, wards, or physicians can be randomised, as well as schools or geographical areas. Such a design is well adapted to assess organisational and behavioural interventions and health promotion programmes—interventions that are usually implemented at the level of health organisational units or geographical areas [9]. As well, randomisation of clusters rather than individuals could prevent contamination. As an example, to assess the impact of a screening programme, widespread publicity is needed to encourage participants to undergo screening. With individual randomisation, communication among participants would induce contamination, but with cluster randomisation, it may strengthen participant compliance.

Use of the CRT has greatly increased over the past 15 years [10] and has motivated the publication of an extension of the CONSORT Statement for this design [11] because of its particular methodological issues. The main issue is that observations from the same cluster are more similar than observations from two different clusters. This situation requires the use of both an inflated sample size and adapted statistical analysis to take into account this concern [6],[7]. Otherwise, for the recruitment process, we can distinguish two different designs: a whole CRT or a CRT with active recruitment. In the first design, once the person in charge of the cluster (the cluster guardian [12]) has agreed to participate, every individual belonging to the cluster is automatically recruited for the trial; in the second design, the guardian (or someone other than the guardian but depending on the guardian) must select and include participants for the trial. Also, Murray distinguishes between cohort designs, whereby a sample of participants is included, followed up, and assessed repeatedly (as is usually done in individually randomised trials), and repeated cross-sectional designs, whereby a distinct sample of participants is assessed at each assessment time [7]. Such a distinction makes sense only in whole CRTs. The design is always a cohort one when it involves active recruitment.

## The Challenge of Comparability of Groups in CRTs

In individually randomised trials, randomisation and allocation concealment aim to prevent selection bias and allow for the comparability of groups at the beginning of the study. The aim of ITT analysis is to maintain this comparability during analysis. In CRTs, the situation is more complex because (i) the design entails a hierarchical structure (cluster and individual levels) and (ii) recruitment and follow-up processes differ from those in individually randomised trials.

First, the hierarchical structure of CRTs requires the consideration of both the cluster and individual levels. When Elbourne et al. [13] appealed for an extension of the CONSORT Statement to CRTs, they explicitly stated that authors must report whether “[the ITT] principle applies to clusters, individuals or both”. Campbell defined ITT analysis in CRTs as taking into account all participants, regardless of whether non-adherence to the protocol occurred at the individual or cluster level [14]. Indeed, in CRTs, participants lost to follow-up and protocol deviation can occur at the level of the cluster (cluster withdrawal or lost to follow-up, inactive cluster, merging of clusters) or the individual (participant withdrawal or lost to follow-up, or transfer from one cluster to another). Moreover, results of a CRT can be analysed by considering the individual or cluster as the statistical unit. Even in the latter case, a summary statistic is estimated from individual responses within each cluster. Therefore, the comparability of groups must be achieved at both the cluster (i.e., for randomised units) and individual levels.

**Figure 1:**
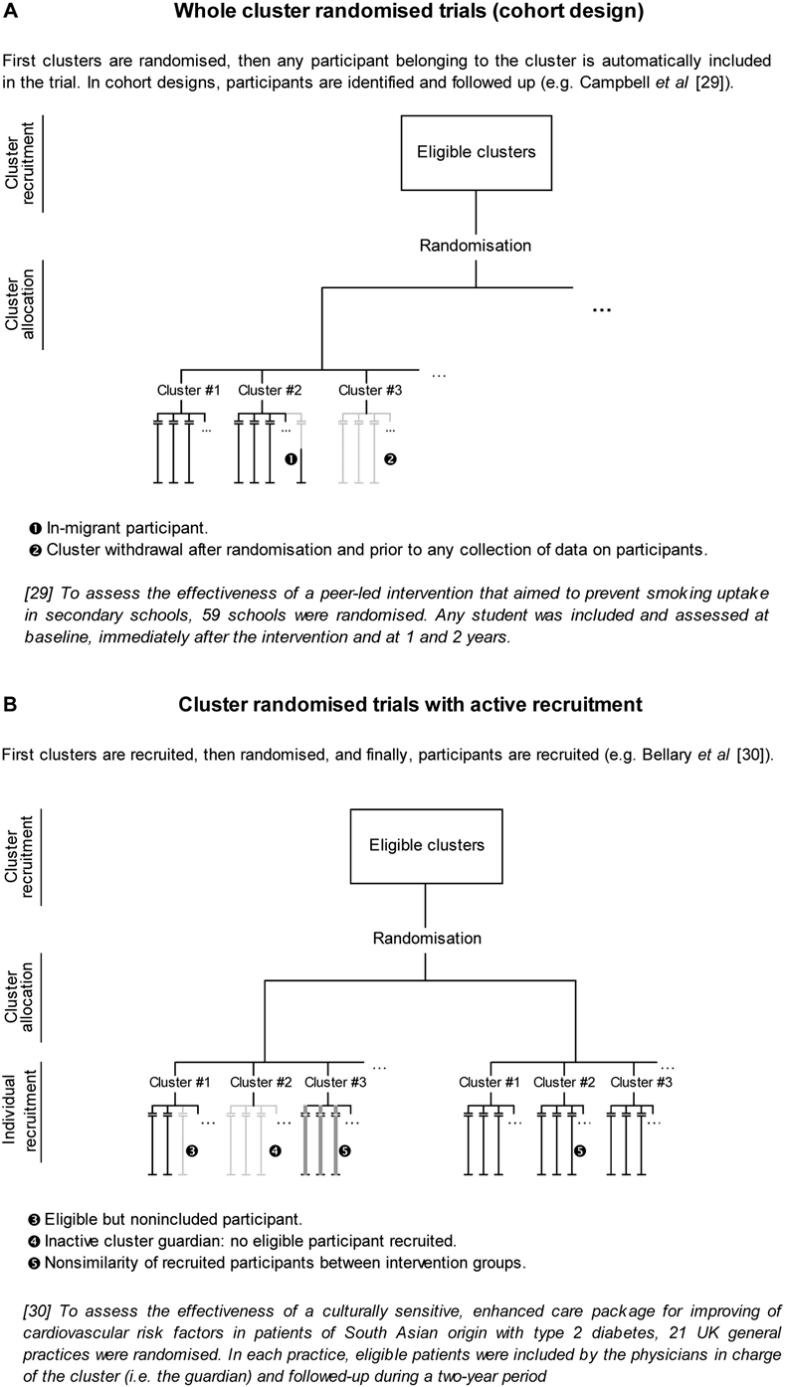
Recruitment issues in cluster randomised trials.

**Figure 2:**
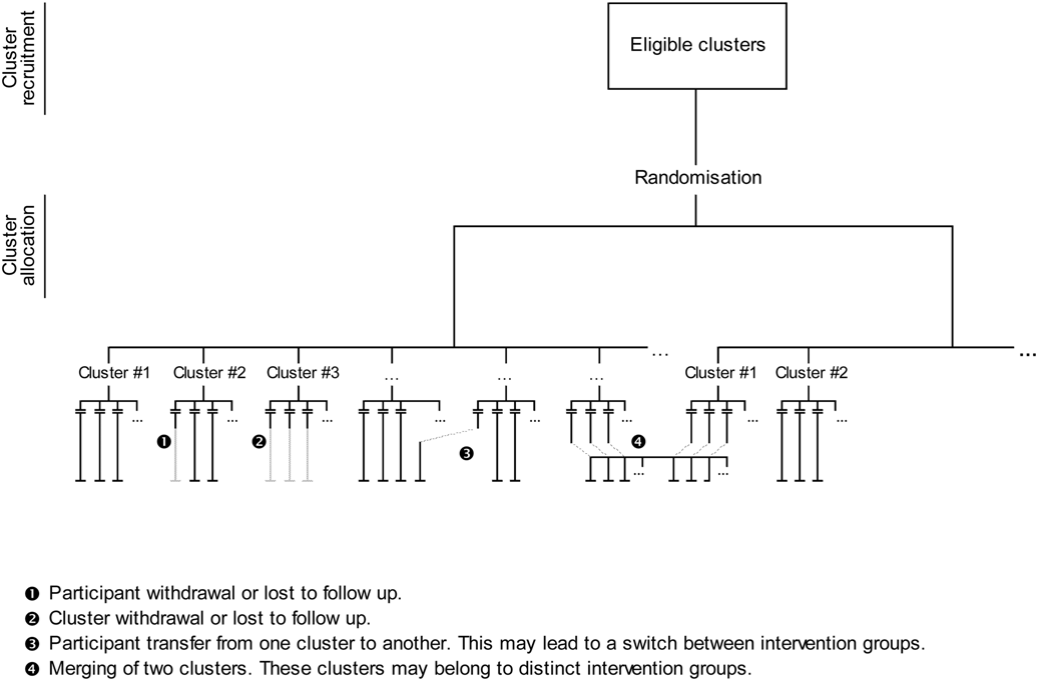
Follow-up issues in cluster randomised trials.

Second, in a CRT, both recruitment and follow-up raise specific issues that may compromise the comparability of groups (Figures 1 and 2). The usual definition of an ITT analysis in CRTs is restrictive because it focuses only on follow-up issues. However, during the recruitment process, clusters may withdraw, or cluster guardians may not actively recruit participants; these situations lead to empty clusters, which should be taken into account to apply the ITT principle. Another important issue is differential recruitment between active clusters, which could be both quantitative (i.e., different number of participants recruited) and qualitative (i.e., participants with different characteristics recruited in both groups). As an example, in the UK BEAM trial, 71.4% of recruited participants were in the intervention group, even though the randomisation was 1∶1, and groups were not comparable on several clinically important criteria [15]. Eldridge et al. concluded that “about a quarter [of CRTs] were potentially biased because of procedures surrounding recruitment and identification of patients” [16], as previously acknowledged by Puffer et al. [8].

## The Challenge of Applying ITT Analysis in CRTs

In CRTs, cluster withdrawal or loss to follow-up and participant withdrawal or loss to follow-up must be handled as is usually done in individually randomised trials, by use of ad hoc missing data methods [17],[18]. More specific to CRTs are participant switches from one cluster to another (e.g., patients who change physicians) and cluster merging (e.g., two medical practices that merge). These follow-up issues are not problematic per se and can be easily handled by keeping clusters as they were randomised. Thus, participants who switch from one cluster to another must be kept in their original cluster, and merged clusters must be split.

The major difference between individually randomised trials and CRTs in applying the ITT principle is that in the former, we know which participants were randomised and must be taken into account in the ITT analysis, whereas in CRTs, we know which clusters have been randomised but we frequently do not know exactly which participants within clusters should have been included in the trial. Whole CRTs and CRTs with active recruitment raise distinct issues, but for both, this dilemma is the real challenge of applying ITT analysis and preserving the comparability of groups.

### Whole CRTs

In whole CRTs, empty clusters occur when cluster guardians withdraw just after the randomisation and before any data on participants are collected. The guardians make these decisions once they know the allocation result, which could influence the likelihood of withdrawal. So excluding such empty clusters from analysis (as is always done) is in essence post-randomisation exclusion. Furthermore, in CRTs, the number of randomised units is much lower than that in individually randomised trials, so any randomised unit has greater influence in a CRT than in an individually randomised trial. Also, in CRTs with empty clusters, although we know some cluster characteristics, often we do not know the number or characteristics of participants who should have been included in those clusters if they had been active. This scenario prevents the use of any statistical solution: knowing cluster characteristics does not allow in any way the derivation of participant characteristics. Such randomised clusters that lack any data on participants are then removed before analysis, which violates the very definition of the ITT (i.e., exclusion of randomised units). In CRTs, therefore, extreme vigilance is needed to ensure cluster guardians' adherence to the trial protocol, before the randomisation of the clusters they are in charge of, because currently, we lack statistical methods to limit the induced bias.

### CRTs with Active Recruitment

As emphasised, CRTs with active recruitment “first recruit the clusters, then randomise and finally recruit the participants: such an approach invites bias” [19]. In the extreme, some cluster guardians may be inactive (i.e., they do not recruit any participants), and these inactive guardians may moreover be more numerous in one group than in another. This situation is similar to “passive withdrawal”: guardians are inactive recruiters because they do not adhere to the allocation result (i.e., the cluster they are in charge of has not been randomised to the group they expected to be in). Actually, any time recruitment of participants occurs after randomisation, (i) participants are selected by someone who could be aware of the group to which the participants will be allocated, and (ii) participants consent not to random allocation but rather to participation in and allocation to a pre-determined group, which could induce differential recruitment or consent and selection bias [8],[20],[21]. The only way to prevent differential recruitment is to ensure some form of allocation concealment during recruitment. Puffer et al. [8] proposed the identification and complete inclusion of participants before the randomisation of a cluster, which allows for maintenance of the usual chronology of a randomised trial. Such a strategy thus prevents both empty randomised clusters and selection bias but cannot be systematically implemented for logistical reasons (e.g., in a trial including incident patients rather than prevalent cases). Two complementary solutions are (i) changing the time of randomisation by randomising clusters only when the first participant is identified (the index case concept) and (ii) blinding independent recruiters to the allocation group. The index case concept could prevent empty clusters but does not prevent differential recruitment.

Actually, some similarity exists between this potential differential recruitment in CRTs and survey non-response. Non-response is a source of bias because non-responders are not a random sample of surveyed people. In CRTs, participants included without allocation concealment are not a random sample of eligible participants. In the context of survey non-response, much work has been done to calibrate estimation, and methods such as weighting adjustments or imputations are classically used [22],[23]. In CRTs, the situation is more complex because contrary to surveys, the number of eligible participants is not known. However, the question remains as to whether all CRTs really benefit from randomisation and whether some trial results would be better analysed as observational studies with some form of adjustment used, such as propensity scores [24],[25]. Some methodologists have resorted to such a solution [26],[27], which raises another issue: “how the levels of the individual subject and the cluster should be considered in the estimation and application of propensity scores” [28]. Further statistical research to transpose such methods specifically to CRTs is therefore warranted.

We provide some recommendations for the planning and analysis of data in CRTs to improve the comparability of groups in such trials in light of the issues of recruitment and ITT analysis (Box 1): some issues may be easily handled, whereas others remain without a solution.

**Box 1: Recommendations for the Planning of and Analysis of Data in CRTs To Improve the Comparability of Groups**

**Handling Recruitment Issues**

**Cluster level:**

- Monitor cluster guardians' adherence to the protocol before the randomisation of the cluster they are in charge of.
  - For whole CRTs, this monitoring will help prevent clusters withdrawing after randomisation and before the collection of data for participants.
  - For CRTs with active recruitment, this monitoring will help prevent empty clusters due to inactivity of guardians.

**Individual level:**

***Whole CRTs***

- Ensure the inclusion of all participants belonging to the randomised clusters.
- For a cohort design (i.e., participants are included, followed up, and assessed):
  - Consider participants who migrate out of clusters (out-migrants) as being withdrawn or lost to follow-up.
  - Discard data for participants who migrate into a cluster (in-migrants).

***CRTs with active recruitment***

- Whenever possible, identify and completely include participants before randomising a cluster, to maintain the usual chronology of a randomised trial (recruitment followed by randomisation) and help prevent selection bias.

If complete inclusion of participants is not possible, the following two solutions can be used in combination:

- If possible, randomise clusters only when the first participant is included (index case concept) to prevent empty clusters.
- If possible, have blinded independent recruiters include participants, so that the inclusion process can be independent from the allocation process.

**Analysis: Handling Follow-Up Issues**

**Cluster level:**

- Take into account any cluster that withdrew or was lost to follow-up by using ad hoc missing data methods for participants included in these clusters.
- If two or more clusters merge during the trial, split the merged clusters so that the clusters analysed are those that were initially recruited.

**Individual level:**

- Take into account any participant who withdrew or was lost to follow-up by using ad hoc missing data methods.
- Keep any participant who transferred from one cluster to another in the original cluster.

**Analysis: Handling Quantitative and Qualitative Imbalance between Intervention Groups**

**Whole CRTs:**

- Use adjustment methods to cope with potential imbalances in both cluster and individual characteristics, which are frequent when the number of randomised clusters is small.

**CRTs with active recruitment:**

- Calibrate estimation by using weighting adjustments (e.g., propensity-score methods) to deal with quantitative and qualitative imbalance due to potential differential recruitment induced by lack of allocation concealment when including participants.

## Conclusion

The application of the ITT principle in analysis of data is much more complex in CRTs than in individually randomised trials because the principle must be applied at the level of both the cluster and the individual, and because of challenging issues surrounding the recruitment process in CRTs. These issues raise concerns regarding the internal validity of such trials.

## Abbreviations

CRT: cluster randomised trial
ITT: intention to treat
